# Screening for efficient nitrogen sources for overproduction of the biomass of the functionally probiotic *L. plantarum* strain RPR42 in a cane molasses-based medium

**DOI:** 10.1186/s13568-020-00976-x

**Published:** 2020-03-17

**Authors:** Moslem Papizadeh, Mahdi Rohani, Seyed Nezamedin Hosseini, Seyed Abbas Shojaosadati, Hossein Nahrevanian, Malihe Talebi, Mohammad Reza Pourshafie

**Affiliations:** 1 Department of Microbiology, Pasteur Institute of Iran, Tehran, Iran; 2 Department of Recombinant Hepatitis B, Vaccine, Production and Research Complex, Pasteur Institute of Iran, Tehran, Iran; 3 Department of Biotechnology Group, Chemical Engineering Department, Tarbiat-Modares University, Tehran, Iran; 4 Department of Parasitology, Pasteur Institute of Iran, Tehran, Iran; 5 Microbial Biotechnology Research Center, Department of Microbiology, School of Medicine, Iran University of Medical Sciences, Tehran, Iran

**Keywords:** Cane molasses, Industrial culture media, *L. plantarum*, Response surface methodology, Probiotics

## Abstract

Nitrogen source has a vital role for the efficient growth of lactobacilli. The effects of cheese whey, corn steep liquor, and wheat germ extract on the growth of *L. plantarum* strain RPR42 in cane molasses-based media was evaluated using various approaches of design of experiments. Our results showed that such protein-rich agricultural by-products significantly increase the biomass production of the strain RPR42 in cane molasses-based media. The most affecting nitrogenous material was cheese whey followed by CSL and the minor effect was reported for wheat germ extract as revealed in factorial and Box–Behnken design experiments. The replacement of costly beef extract and yeast extract with a defined mixtures of the above nitrogenous agricultural by-products in cane molasses-based medium led to production of up to 12.64 g/L/24 h of dry biomass of strain RPR42. A detectable cell density of strain RPR42 (~ 9.81 × 10^9^ CFU/mL 24 h) which was observed in such an economic medium showed that the large-scale production of the strain RPR42 tend to be feasible at significantly low costs.

## Introduction

Functional foods, containing beneficial strains of microbes which belong mainly to lactic acid bacteria, have recently made a global revolution in the food market (Stanton et al. 2001; Khan and Ansari 2007). Probiotics, as a characteristic component of functional foods, are viable microorganisms that have beneficial effects on the health through some adjustments in the microbiota when consumed in adequate amounts (Papizadeh et al. 2016). Hence, they have been recently suggested as an alternative to antibiotics in animals and humans (Tiwari et al. 2012) and consequently, a growing market is expected for probiotics and functional foods, worldwide (Khan and Ansari 2007).

The industrial production of probiotics at low costs has been focused since the large scale production of functional foods incorporated with probiotics has become popular. The importance of more efficient media and bioprocesses have therefore been highlighted (Yoo et al. 2018). Introduction of an optimized culture medium for biomass production of a given strain depends mainly on the physiological properties of the target strain. Considering the fact that the main probiotic genera are *Bifidobacterium* and *Lactobacillus* (Papizadeh et al. 2017; Papizadeh and Pourshafie 2017), an optimized medium for industrial cultivation of these bacteria could be expensive, requiring costly and complex supplements (peptones, beef extract, casein hydrolysate, and yeast extract) (Manzoor et al. 2017). Furthermore, such microbes have unique physiological properties (Wee et al. 2004) which caused their characteristic low growth yield. De Man, Rogosa and Sharpe (MRS) broth is a very rich and expensive medium which is routinely used for cultivation of various *Lactobacillus* strains (Horn et al. 2005). Considering the growth requirements of lactobacilli, costly complex supplements are used for laboratory-scale cultivation of them. Hence, the probability of the replacement of such expensive supplements with efficient and economic alternatives has been a bottleneck for commercial overproduction of the biomass of the probiotic lactobacilli (Hwang et al. 2012).

Molasses has been introduced as an efficient carbon source for large-scale production of various microbial strains (Barbosa et al. 2016; Okafor and Okeke 2017). However, the insignificant organic nitrogen content of molasses necessitates the addition of nitrogen sources for growth improvement of microbial strains in molasses-based media (Lino et al. 2018).

The corn steep liquor (CSL), an agricultural by-product which is produced through the corn milling process, has been widely used as an economic nutrient-rich constituent of culture media in various large-scale bioprocesses (Manzoor et al. 2017). CSL contains significant contents of amino acids, polypeptides and B-group vitamins and so, is a recommended nitrogen source for a diverse array of bioprocesses (Cardinal and Hedrick 1948). Another nitrogen-rich agricultural by-product which has been widely used in biotechnology is cheese whey which is a main by-product of dairy industry. Cheese whey contains about 55% of the nutrients of the milk and is a recommended carbon source since it has a considerable lactose content (Manzoor et al. 2017). This by-product also contains significant fractions of soluble proteins (0.6–0.8% w/v), lipids and minerals (Manzoor et al. 2017). Considering the aforementioned contents of the cheese whey, it can be also used as a whole culture medium for cultivation of some microbial species (Manzoor et al. 2017; Okafor and Okeke 2017). Wheat germ extract (WGE), is another nutrient-rich substrate which can be regarded as a product of cereal agriculture. Despite the nutritional qualities which has been well-documented for fermented WGE by yeasts and LAB strains, this substrate have been barely used for biomass production of microbial strains (Verni et al. 2019). However, in spite of the economic importance of the above mentioned agricultural nutrient rich substrates, the major portion of them is discarded in the environment as wastes which will have its own environmental pollution consequences due to the high biochemical oxygen demands (BOD).

Many studies have tried medium optimization for increased biomass production of lactobacilli (Horn et al. 2005; Krzywonos and Eberhard 2011; Hwang et al. 2012; Yoo et al. 2018), however, the medium optimization for lactobacilli have mainly aimed the production of bacteriocins (Barman et al. 2018), exopolysaccharides (Benhadria et al. 2017) and lactic acid (Srivastava et al. 2015).

The aim of this research was to improve the efficiency of a cane-molasses based medium for overproduction of biomass of *Lactobacillus plantarum* strain RPR42 on least possible expenses. Thus, the efficiency of CSL, wheat germ extract (WGE), and cheese whey, as economic nitrogenous substrates was studied to optimize an economic medium for overproduction of the biomass of strain RPR42. Then, the statistical optimization of the medium formula was performed using varied approaches of design of experiment (DOE).

## Materials and methods

Cane molasses was obtained from Dehkhoda sugarcane Agro-industry Company, Ahvaz, Iran. Minerals were purchased from Merck. CSL was kindly provided by faculty of biological sciences, Alzahra University, Tehran, Iran. Cheese whey powder was purchased from BornaLaban Dairy Products. Beef extract, yeast extract and casein hydrolysate were obtained from the Microbiology Department of Pasteur Institute of Iran.

### Microbial strain

*Lactobacillus plantarum* strain RPR42, accessible from the Culture Collection at Pasteur Institute of Iran under the accession code RPR42, is a functionally probiotic lactobacilli which was isolated from human fecal samples. The probiotic properties of strain RPR42 have been summarized, previously (Rohani et al. 2015, 2018). Aliquots of the strain RPR42 cell suspension (~ 1.3 × 10^9^ CFU/mL) were preserved in 20% (w/v) glycerol solution at − 80 °C.

### Inoculations

A preserved cell suspension of the strain RPR42 was incubated at 55 °C for a minute to facilitate the rapid thawing. Then, 50 mL of auto-clave-sterilized MRS medium (Merck) was inoculated by an aliquot (100 µL) of the thawed cell suspension in a 60 mL glass bottle. Incubation of the culture was performed at 37 °C (the preferred temperature for the growth of strain RPR42) for 24 h and then 8-cm plates of MRS agar medium were streaked by a 50 µL of this culture for quality control of the purity. For inoculation of the screening and either production media an inoculum size of 10% (v/v) was considered. The starting optical density of the inoculated cultures was ~ 0.12 ± 0.005, corresponding to ~ 1.75 × 10^7^ CFU/mL (Krzywonos and Eberhard 2011; Nancib et al. 2015).

### Optimization of the medium

#### Single variable at a time experiments

The preliminary screenings assessed the reaction of the strain RPR42 to a gradient of concentrations of CSL [5, 7.5, 10, 12.5, 15, 17.5, and 20/% (v/v)], WGE [55, 60, 70, 75, 80, 90, and 100% (v/v)], and cheese whey [0.5, 1, 2.5, 5, 7.5, 10, and 12.5% (w/v)]. All screening experiments were performed in 20 mL glass bottles (sealed with aluminum rings and rubber snap caps) with final volume of 15 mL. Also, all cultures of the screening experiments were incubated at 37 °C in static conditions. All experiments were conducted aseptically in triplicate and average values were calculated.

#### Factorial design

Biomass production was optimized through multiple 4^2^ factorial designs with two independent factors, including cane molasses as the carbon source, and three different nitrogenous substrates. Factorial design 1 used CSL as the nitrogen source at four concentrations [7.5, 10, 12.5 and 15% (v/v)]. Factorial design 2 used WGE as a nitrogen source at four concentrations [40, 50, 60 and 70% (v/v)]. Factorial design 3 used cheese whey as a nitrogen source at four concentrations [0.5, 2.5, 5 and 10% (w/v)]. In the above factorial designs cane molasses was used as the carbon source at four concentrations [12.5, 15, 20, and 25% (v/v)] (Additional file 1: Table S1). Also, additional sets of experiments were regarded considering the observed results in the above factorial designs. The experimental designs are shown in Additional file 1: Table S1. In each factorial design, firstly, 16 duplicate experiments and so, totally 48 experiments were performed in duplicate. However, in case of cheese whey, 12 additional duplicate experiments were performed to find the optimal formula of cane molasses + cheese whey (Additional file 1: Table S1). The measured response variable was biomass production, which was expressed as dry biomass (g/L) (Lim et al. 2018; Pérez-Armendáriz et al. 2019).

#### Response surface methodology

As the next step, the response surface methodology (RSM) was conducted by applying Box–Behnken design and central composite design (CCD) to confirm the optimal medium formulation for biomass production and also to decipher the interactions between the factors (Liew et al. 2005; Myers et al. 2009). The factors and the corresponding levels of Box–Behnken design and central composite design are shown in Tables 1 and 3, respectively. Different regression models were used to interpret the data. Interactions between various couples of the factors and the corresponding effects on the biomass production were analyzed by observing the contour response curves which were generated. The lack of fit was used for controlling the statistics adequacy and efficiency of the model.

**Table 1:** Box–Behnken design of experiment using four variables (A, cane molasses; B, CSL; C, WGE; D, and cheese whey)

Std	Run	Factor (A): cane molasses [% (v/v)]	Factor (B): CSL [% (v/v)]	Factor (C): WGE [% (v/v)]	Factor (D): cheese whey [% (w/v)]	Dry biomass (g/L)
3	1	10	7.5	35	3	9.34
13	2	12.5	3	10	3	9.19
9	3	10	5.25	35	1	9.5
15	4	12.5	3	70	3	9.17
24	5	12.5	7.5	35	5	7.91
8	6	12.5	5.25	70	5	8.21
12	7	15	5.25	35	5	7.59
28	8	12.5	5.25	35	3	9.12
2	9	15	3	35	3	8.45
17	10	10	5.25	10	3	9.7
25	11	12.5	5.25	35	3	9.12
11	12	10	5.25	35	5	9.1
6	13	12.5	5.25	70	1	8.94
23	14	12.5	3	35	5	8.51
18	15	15	5.25	10	3	8.41
26	16	12.5	5.25	35	3	9.15
5	17	12.5	5.25	10	1	9.08
14	18	12.5	7.5	10	3	8.82
20	19	15	5.25	70	3	8.26
7	20	12.5	5.25	10	5	8.35
10	21	15	5.25	35	1	8.57
19	22	10	5.25	70	3	9.59
22	23	12.5	7.5	35	1	8.88
27	24	12.5	5.25	35	3	9.11
16	25	12.5	7.5	70	3	8.56
4	26	15	7.5	35	3	8.07
1	27	10	3	35	3	9.81
21	28	12.5	3	35	1	9.01

The averages for the triplicate dry biomass weights are presented and finally values rounded to the nearest 0.00–0.09

#### Analytical methods

The optical density of cultures was measured using a UV–visible spectrophotometer (Shimadzu Co, Tokyo, Japan). Samples of the culture (1 mL), taken at 2-h intervals, centrifuged at 8000 rpm (7168 RCF) for 5 min (Beckman Coulter, Allegra™ X-22R Centrifuge), double washed (0.96% saline buffer) and finally suspended in 1 mL of the same buffer. The absorbance of such cell suspensions was measured at 600 nm (OD_600_). Dilution of the samples (2–20-times) with 0.96% saline buffer was performed in case of > 0.4 absorbance values. Also, 5 mL samples of the cultures were passed through a filter paper and the cells were double-washed with 0.96% saline buffer. The cells were dried at 60 °C for 24 h in an oven and finally weighed to constant weight. According to the established regression between the dry cell weight and OD_600_, the biomass was expressed as g/L of dry biomass. Simultaneously, the viable cells were counted in selected cultures using serial dilution (10 to 10^9^) and plating techniques. Serial dilutions prior to plating were performed on ice. For plating, 8-cm plates of MRS agar were inoculated by 50 µL of the 10^7^, 10^8^, 10^9^ and 10^10^ dilutions followed by homogenously streaking. Finally, 48 h incubation of the plates was performed at 37 °C prior to enumeration of the colony-forming units.

#### Statistical analysis of data

The statistical significances were analyzed using analysis of variance (ANOVA). The response surface methodology (RSM) experiments were performed using the Design Expert package (v10.0.4.0.x64, Stat-Ease Inc).

## Results

### Single variable at a time experiments

Strain RPR42 grew well in various concentrations of cheese whey, CSL, and WGE (Additional file 1: Figures S1–S3). The optimal concentration of the CSL for the growth of strain RPR42 was 10% (v/v) in which 3.63 g/L of dry biomass of strain RPR42 was produced (Additional file 1: Figure S1). In place, our preliminary investigations indicated that the best growth efficiency of strain RPR42 in cane molasses, 4.12 g/L of dry biomass, can be observed in 22.5% (v/v) although it still grew well in 17.5–25% (v/v) solutions of cane molasses (data not shown). The growth of strain RPR42 in various concentrations of WGE was examined and the results showed that the largest biomass, 3.84 g/L of dry biomass, was recorded in the 70% (v/v) solution (Additional file 1: Figure S2). In comparison, strain RPR42 produced significant biomass in 2.5–12.5% (w/v) solutions of cheese whey, but its optimal growth, 4.16 g/L/24 h of dry biomass, was recorded in 7.5% (w/v) solution of this substrate (Additional file 1: Figure S3).

### Factorial design experiments

**Fig. 1:**
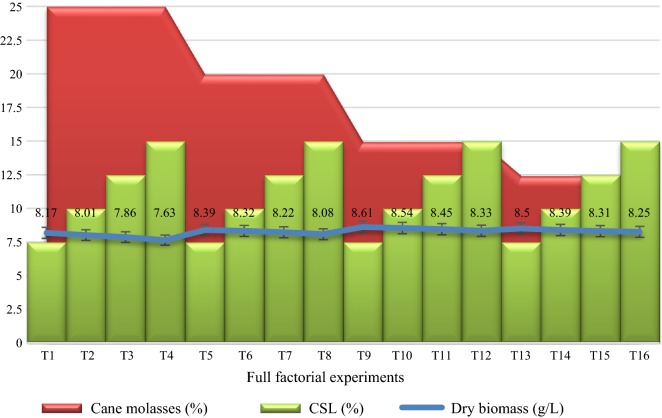
The full 2^4^ factorial design used for optimization of the biomass production of strain RPR42 in various concentrations of cane molasses as the carbon source versus CSL as the nitrogen source (Additional file 1: Table S1). The averages for the triplicate dry biomass weights are presented and finally values rounded to the nearest 0.00–0.09

**Fig. 2:**
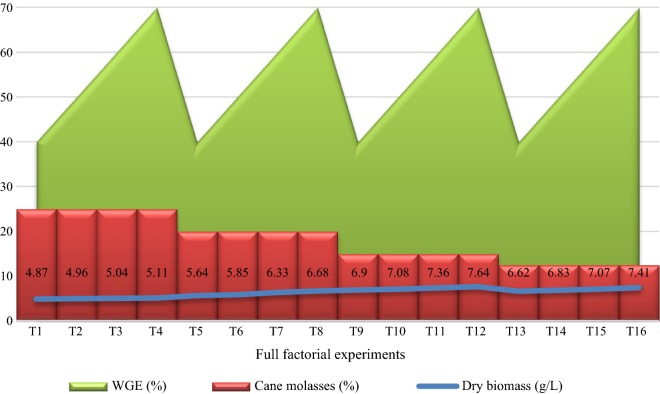
The full 2^4^ factorial design used for optimization of the biomass production of strain RPR42 in various concentrations of cane molasses as the carbon source versus WGE as the nitrogen source (Additional file 1: Table S1). The averages for the triplicate dry biomass weights are presented and finally values rounded to the nearest 0.00–0.09

**Fig. 3:**
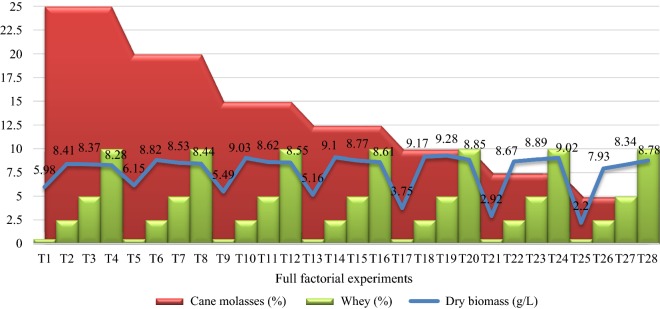
Full factorial designs (Factorial design A: T1–T16, Factorial design B: T17–T32) used for optimization of the biomass production of strain RPR42 in various concentrations of cane molasses as the carbon source versus cheese whey as the nitrogen source (Additional file 1: Table S1). The averages for the triplicate dry biomass weights are presented and finally values rounded to the nearest 0.00–0.09

Factorial design experiments revealed the interactions between the cane molasses (the main carbon source) and the three nitrogenous substrates (CSL, WGE, and cheese whey). Results of the factorial design 1 (Additional file 1: Table S1) showed that a maximal 8.61 g/L of dry biomass of strain RPR42 was obtainable when the medium contained 15% and 7.5% (v/v) cane molasses and CSL, respectively (Additional file 1: Table S1, Fig. 1). In place, the results of the factorial design 2 (Additional file 1: Table S1) indicated that the largest biomass yield, 7.64 g/L of dry biomass, can be produced in the presence of 10% (v/v) cane molasses and 70% (v/v) WGE (Additional file 1: Table S1, Fig. 2). The factorial design 3 indicated that a solution containing 10% (v/v) cane molasses and 5% cheese whey (w/v) could produce the largest biomass yield, 9.28 g/L of dry biomass (Additional file 1: Table S1, Fig. 3).

According to the results obtained from above factorial design experiments, the influence of various combinations and concentrations of polysorbates (Tween 20, 40, 60, and 80) on the biomass production of strain RPR42 was assessed using previous formulations. It was found that the optimal polysorbate concentration in cane molasses-based media containing CSL, WGE, and cheese whey was 0.075%, 0.06–0.065%, and 0.05–0.065%, respectively. In comparison, based on our preliminary findings (data not shown), the optimal polysorbate concentration for growth of strain RPR42 in solutions containing only cane molasses was 0.075% (v/v). Also, the addition of a 1:1:1 mixture of Tween 20, 60, and 80 into the cane molasses [10% (v/v)] + CSL [7.5% (v/v)] medium resulted in 8.85 g/L/24 h of dry biomass (Additional file 1: Figures S4 and S5). Similarly, the above combined mixture of polysorbates enhanced the biomass production in cane molasses-based media containing WGE (7.8 g/L/24 h of dry biomass) (Additional file 1: Figures S6 and S7) or cheese whey (9.33 g/L/24 h of dry biomass) (Additional file 1: Figures S8 and S9). Furthermore, the single variable at a time experiments showed that the mostly favored polysorbate for the growth of strain RPR42 in various cane molasses-based media is Tween 80 followed by Tween 20, 60 and 40, respectively (Additional file 1: Figures S4–S9).

**Fig. 4:**
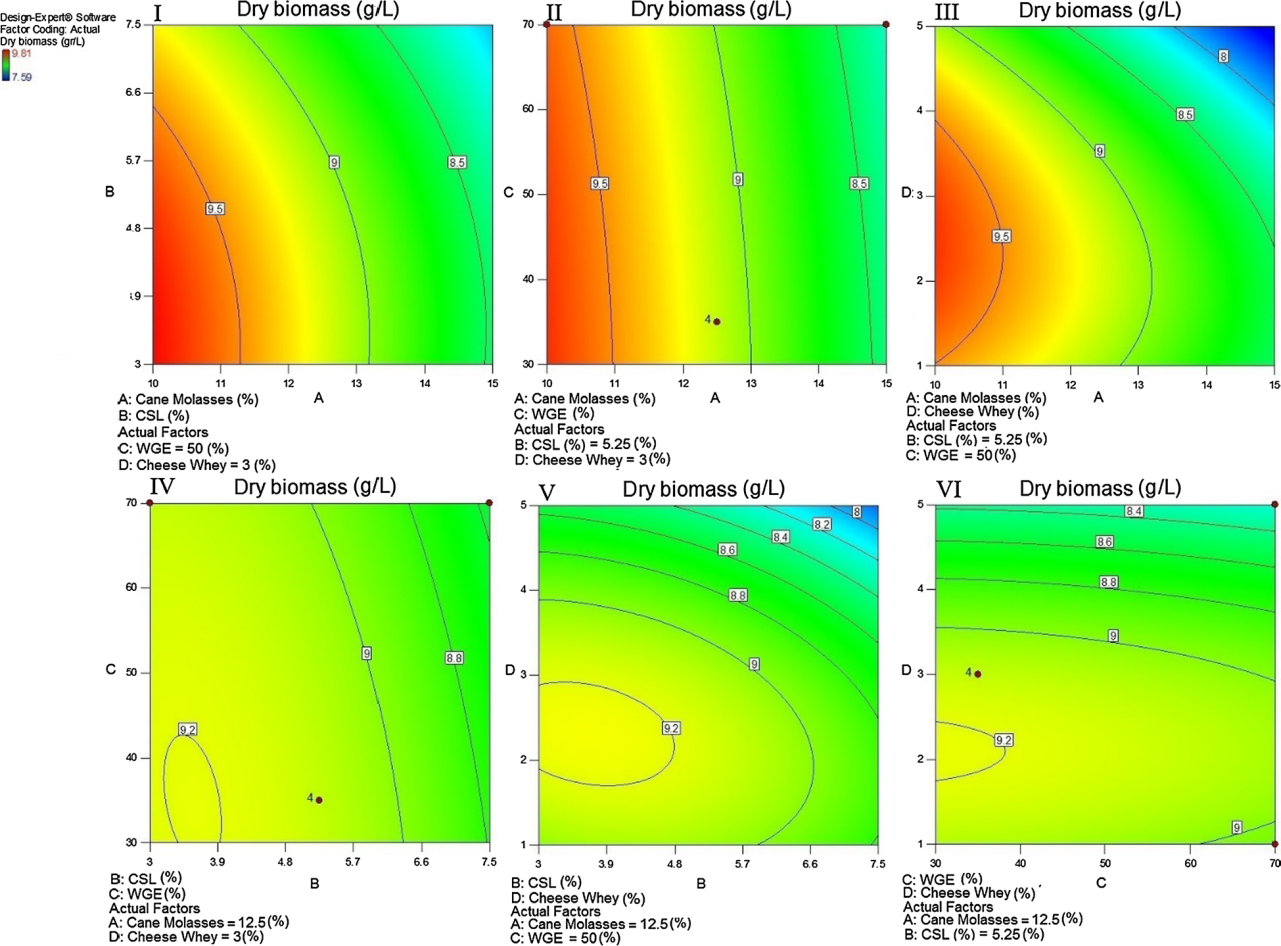
Contour plot surface of dry biomass production of strain RPR42 showing the interactions between cane molasses and CSL; I, cane molasses and WGE; II, cane molasses and cheese whey; III, CSL and WGE; IV, CSL and cheese whey; V, WGE and cheese whey; VI, in a Box–Behnken design

Then, the probable effectiveness of a combined mixture of CSL, WGE, and cheese whey in cane molasses solutions was preliminary evaluated using the Box–Behnken design (Table 1). A 1:1:1 mixture of Tween 20, Tween 60, and Tween 80 with total polysorbate concentration of 0.07% (v/v) was also added into all experiments. The interaction of the aforementioned variables and the optimum level of each variable for a maximum response were illustrated as contour plots (Fig. 4). Various combinations of each couple of the test variables were depicted as different response surfaces (Fig. 4). According to the results of Box–Behnken design experiment, cane molasses and cheese whey significantly affected the biomass production of the strain RPR42. However, CSL and WGE had lower impact on the above response (Tables 1 and 2, Fig. 4).

**Table 2:** Analysis of variance (ANOVA) for the response surface of full quadratic model for optimization of four categories of additives (A, cane molasses; B, CSL; C, WGE; D, cheese whey)

Source	Sum of squares	Degree of freedom	Mean square	F value	p-value Prob > F
Model	8.22	14	0.59	424.22	< 0.0001
A-cane molasses	3.17	1	3.17	2287.67	< 0.0001
B-CSL	0.44	1	0.44	320.33	< 0.0001
C-WGE	0.082	1	0.082	59.17	< 0.0001
D-cheese whey	0.98	1	0.98	708.34	< 0.0001
AB	2.025E−003	1	2.025E−003	1.46	0.2481
AC	6.001E−004	1	6.001E−004	0.43	0.5219
AD	0.084	1	0.084	60.73	< 0.0001
BC	0.017	1	0.017	12.08	0.0041
BD	0.055	1	0.055	39.88	< 0.0001
CD	3.712E−006	1	3.712E−006	2.681E−003	0.9595
A^2^	0.019	1	0.019	13.71	0.0027
B^2^	0.12	1	0.12	84.92	< 0.0001
C^2^	0.020	1	0.020	14.68	0.0021
D^2^	0.95	1	0.95	688.89	< 0.0001
Residual	0.018	13	1.385E−003		
Lack of fit	0.017	10	1.710E−003	5.70	0.0894

Adj R-squared: 0.9955, Pred R-squared: 0.9879, Adeq precision: 83.541

Final equation in terms of coded factors:

$$\begin{aligned} {\text{Dry biomass}} & = \, + 9.0 9- 0. 6 5 {\text{ A}} - 0. 2 4 {\text{ B}} - 0.0 7 2 {\text{ C}} - 0. 3 6 {\text{ D}} + 0.0 2 3 {\text{ AB}} - 8.0 9 1 {\text{E}} - 00 3 {\text{AC}} - 0. 1 4 {\text{ AD}} \\ & \quad - 0.0 4 3 {\text{ BC}} - 0. 1 2 {\text{ BD}} - 6. 3 6 4 {\text{E}} - 00 4 {\text{ CD}} - 0.0 5 6 {\text{ A}}_{ 2} - 0. 1 4 {\text{ B}}_{ 2} + 0.0 2 7 {\text{ C}}_{ 2} - 0. 40{\text{ D}}_{ 2} \\ \end{aligned}$$

Cane molasses (A), CSL (B), WGE (C), and cheese whey (D), with their corresponding levels as four factors were included in the design experiment and the results obtained from the 28 experiments using Box–Behnken design are summarized in Table 1. The highest cell density production obtained in these screening experiments was 9.81 g/L/24 h of dry biomass as demonstrated in Table 1 (run 27). The quadratic interaction effects were calculated with the screening experiments data.

As demonstrated in Table 2, the model F-value of 424.22 is indicative of the significance of the model. ANOVA results also indicated that all model terms were significant. In addition, the lack of Fit F-value of 5.70 indicated the insignificance of the “Lack of Fit” relative to the pure error. Furthermore, the promising R2 value (0.9978) implies that the closeness of the data to the predicted values from the model. In addition, the “Pred R-Squared” (0.9879) and the “Adj R-Squared” (0.9955) of the model are in a reasonable agreement since their difference is less than 0.2 (Table 3). The ANOVA results of the quadratic response surface model for biomass production are demonstrated in Table 2. The inferior probability value of the model (p-value Prob > F < 0.0001) (Table 2) shows the promisingly significance of the quadratic regression model. According to the results obtained from the Box–Behnken design experiments, the design expert software suggested the best formulation of variable levels as below: 10% (v/v) molasses, 3.5% (v/v) CSL, 38.1% (v/v) WGE, and 2.6% (w/v) cheese whey. The produced biomass in the above formula (9.84 g/L of dry biomass) was comparable to that observed in run 27 of the Box–Behnken design experiments (9.81 g/L of dry biomass) (Table 1).

**Table 3:** Central composite design of experimental response of four variables (A, cane molasses; B, combined nitrogenous mixture; C, casein hydrolysate; D, glucose)

Std	Run	Factor (A): cane molasses [% (v/v)]	Factor (B): combined nitrogenous mixture (%)^a^	Factor (C): casein hydrolysate (g/L)	Factor (D): glucose (g/L)	Dry biomass (g/L)
3	1	8	30	6.5	4.5	11.6
13	2	10	52.5	5	3	11.51
9	3	12	45	3.5	4.5	11.8
15	4	12	30	6.5	4.5	12.18
24	5	12	30	3.5	4.5	11.46
8	6	12	30	3.5	1.5	11.03
12	7	8	45	3.5	1.5	11.61
28	8	12	30	6.5	1.5	12.02
2	9	10	37.5	5	6	11.9
17	10	8	30	3.5	4.5	10.88
25	11	10	37.5	0	3	11.07
11	12	10	37.5	8	3	12.07
6	13	6	37.5	5	3	11.32
23	14	8	45	6.5	4.5	11.78
18	15	8	30	3.5	1.5	10.27
26	16	10	37.5	5	0	11.65
5	17	12	45	6.5	4.5	11.74
14	18	8	45	3.5	4.5	11.71
20	19	8	45	6.5	1.5	11.86
7	20	12	45	3.5	1.5	11.73
10	21	8	30	6.5	1.5	11.38
19	22	10	37.5	5	3	12.13
22	23	10	22.5	5	3	10.64
27	24	10	37.5	5	3	12.13
16	25	12	45	6.5	1.5	11.91
4	26	14	37.5	5	3	12.06
1	27	10	37.5	5	3	12.11
21	28	10	37.5	5	3	12.15

The averages for the triplicate dry biomass weights are presented and finally values rounded to the nearest 0.00–0.09. All the experiments contained a 1:1:1 mixture of Tween 20, 60 and 80 with a final polysorbate concentration of 0.07%

^a^The constant proportions for CSL [% (v/v)], WGE [% (v/v)] and cheese whey [% (w/v)] for preparation of all combined nitrogenous mixtures were 1.35/14.65/1, respectively

The next set of experiments showed that beef extract, casein hydrolysate, and yeast extract further enhance the growth of strain RPR42 in the medium suggested by the Box–Behnken design experiments. However, considering the expenses of beef extract and yeast extract, the optimal concentration of only casein hydrolysate for biomass production of strain RPR42 was preliminary assessed (Additional file 1: Figure S10). The effect of glucose on the growth of strain RPR42 was also evaluated (Additional file 1: Figure S10). A single-level partial factorial design was conducted in which the influence of glucose and casein hydrolysate concentrations on the growth of strain RPR42 using the Box–Behnken design experiments was evaluated (Additional file 1: Table S2). The results showed that both of casein hydrolysate and glucose have considerable effects on the biomass production of strain RPR42 in the aforementioned medium design (Additional file 1: Figure S10). Hence, casein hydrolysate and glucose were found as effective parameters for further optimization of the cane molasses-based medium.

Additional series of factorial designs highlighted the fact that the minerals, including di-ammonium citrate, sodium acetate, K_2_HPO_4_, MgSO_4_, and MnSO_4_ insignificantly increase the biomass production of strain RPR42 in CSL, WGE or cheese whey containing cane molasses-based media. However, K_2_HPO_4_, among the studied minerals, had the strongest effect on the growth of strain RPR42 (Additional file 1: Tables S3–S5). Hence, the minerals were not included in the final optimization experiments.

**Fig. 5:**
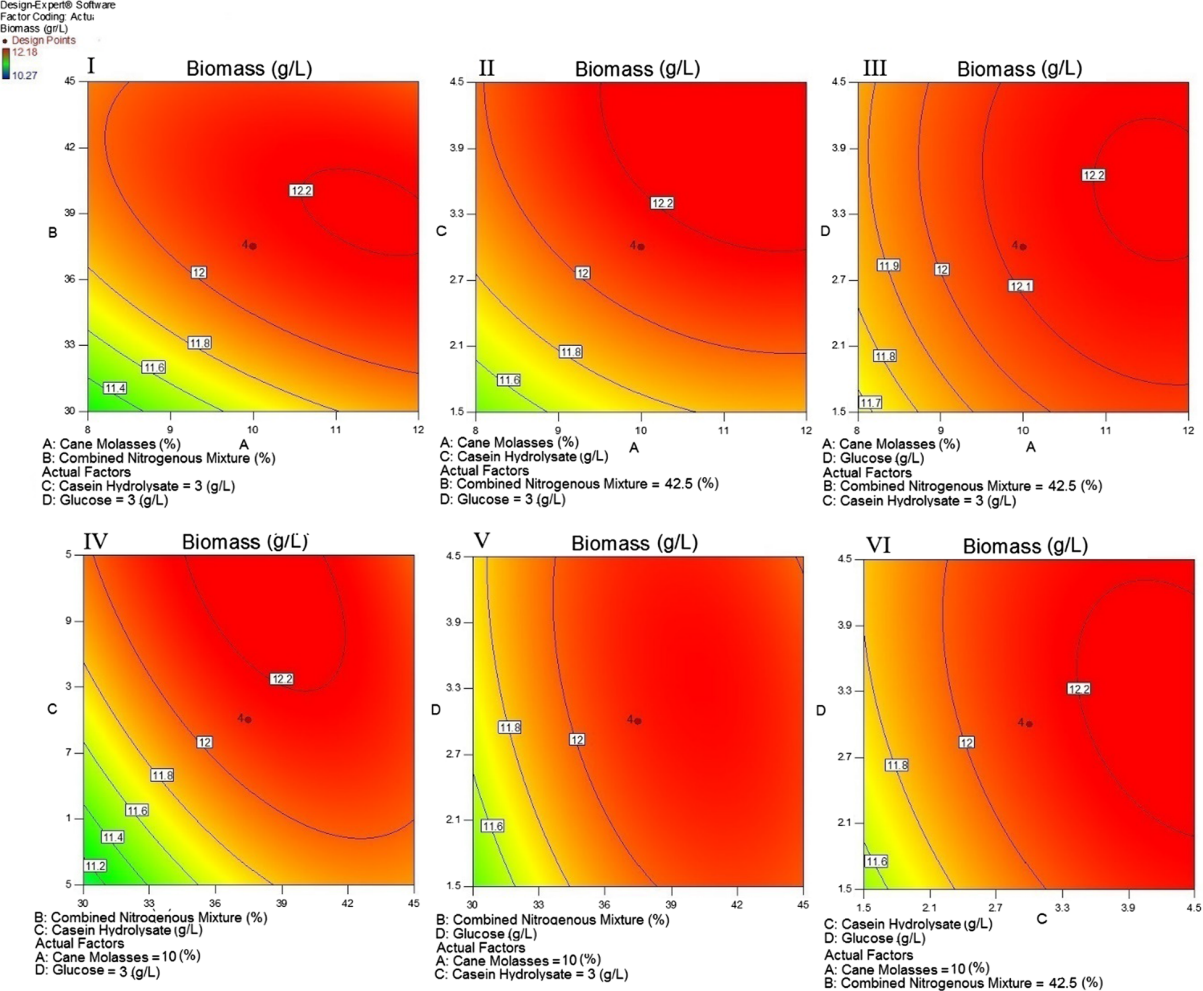
Contour plot surface of dry biomass production by strain RPR42, showing the Interactions between cane molasses and combined nitrogenous mixture; I, cane molasses and casein hydrolysate; II, cane molasses and glucose; III, combined nitrogenous mixture and casein hydrolysate; IV, combined nitrogenous mixture and glucose; V, casein hydrolysate and glucose; VI, in a central composite design

Considering the above findings, cane molasses, casein hydrolysate, glucose, and a combined mixture of CSL, WGE, and cheese whey were used to conduct a central composite design (CCD) for final optimization of the biomass production of strain RPR42 (Table 3). Cane molasses (A), combined nitrogenous mixture, comprising CSL, WGE, and cheese whey (B), casein hydrolysate (C), and glucose (D), with their corresponding levels as four parameters were included in the design experiment (Table 3). All experiments contained a 1:1:1 mixture of Tween 20, Tween 60, and Tween 80 with total polysorbate concentration of 0.07% (v/v). The results obtained from the 28 triplicate experiments using central composite design are demonstrated in Table 3. The interactions between the above variables and their optimal levels for the optimal biomass production were shown as contour response surface plots (Fig. 5).

Final equation in terms of coded factors:

$$\begin{aligned} {\text{Dry biomass}} & = + 1 2. 1 3+ 0. 1 8 {\text{ A}} + 0. 2 1 {\text{ B}} + 0. 2 5 {\text{ C}} + 0.0 7 7 {\text{ D}} - 0. 1 5 {\text{ AB}} - 0.0 20{\text{ AC}} - 0.0 2 2 {\text{ AD }} \\ & \quad - 0. 1 9 {\text{ BC}} - 0.0 9 4 {\text{ BD}} - 0.0 6 7 {\text{ CD}} - 0. 10{\text{ A}}_{ 2} - 0. 2 6 {\text{ B}}_{ 2} - 0. 1 3 {\text{ C}}_{ 2} - 0.0 8 3 {\text{ D}}_{ 2} \\ \end{aligned}$$

Cane molasses, casein hydrolysate, and the combined nitrogenous mixture had significant effects on the biomass production of the strain RPR42 and glucose had the lowest but still significant effect on the growth of strain RPR42 (Table 4, Fig. 5). ANOVA results of the quadratic response surface model for biomass production is shown in Table 4. The Model F-value of 343.73 shows the statistical significance of the model (Table 4). ANOVA results showed that there have been only a 0.01% chance that noise caused such a considerable F-value. The Prob > F values of all the model terms were statistically significant (< 0.05). Furthermore, the lack of Fit F-value was 6.15 which shows the insignificance of the “Lack of Fit” relative to the pure error, statistically. Also, 8.08% chance has been there that noise resulted such a considerable F-value of lack of fit. Such an insignificant F-value of lack of fit of the model have led to a close correlation of the data and the model. Furthermore, the considerable R2 value (0.9973) indicates the data and the predicted values from the model were very close. Also, the signal to noise ratio was 70.379 which indicated an adequate signal and such Adeq precision value mirrored the possibility of design space navigation by the selected model (Table 4).

**Table 4:** Analysis of variance (ANOVA) for the response surface of full quadratic model used for optimization of four categories of additives (A, cane molasses; B, combined nitrogenous mixture; C, casein hydrolysate; D, glucose)

Source	Sum of squares	Degree of freedom	Mean square	F Value	p-value Prob > F
Model	6.37	14	0.46	343.73	< 0.0001
A-cane molasses	0.76	1	0.76	571.23	< 0.0001
B-combined nitrogenous mixture	1.07	1	1.07	805.92	< 0.0001
C-casein hydrolysate	1.49	1	1.49	1125.63	< 0.0001
D-glucose	0.14	1	0.14	106.57	< 0.0001
AB	0.34	1	0.34	258.53	< 0.0001
AC	6.400E−003	1	6.400E−003	4.83	0.0466
AD	8.100E−003	1	8.100E−003	6.12	0.0279
BC	0.60	1	0.60	453.74	< 0.0001
BD	0.14	1	0.14	106.23	< 0.0001
CD	0.073	1	0.073	55.07	< 0.0001
A^2^	0.26	1	0.26	198.31	< 0.0001
B^2^	1.60	1	1.60	1209.98	< 0.0001
C^2^	0.43	1	0.43	328.40	< 0.0001
D^2^	0.17	1	0.17	125.91	< 0.0001
Residual	0.017	13	1.324E−003		
Lack of fit	0.016	10	1.641E−003	6.15	0.0808

Adj R-squared: 0.9944, Pred R-squared: 0.9850, Adeq precision: 70.379

The highest cell density production obtained in the CCD experiments was ~ 9.54 × 10^9^ CFU/mL, corresponding to 12.11 up to 12.18 g/L of dry biomass 24 h (Table 3) which was observed in runs 4, 22, 24, 27, and 28. The quadratic interaction effects were calculated with the optimization experiments data. Also, the design expert software suggested the optimal formulation of the studied variable levels as below: 9.663% (v/v) cane molasses, 4.42% of the combined nitrogenous mixture, 4.19 g/L casein hydrolysate, and 2.6 g/L glucose. The produced biomass in this formula (12.19 g/L of dry biomass) was comparable to those observed in runs 4, 22, 24, 27, and 28 of the CCD experiments (Table 3). Also, adjustment of the pH at 6.8, which was the preferred pH for the growth of strain RPR42, increased the produced biomass in the above medium up to 12.64 g/L (corresponding to ~ 9.81 × 10^9^ CFU/mL).

## Discussion

It has been previously indicated that the cane molasses-based media can be used for overproduction of the biomass of the LAB strains (Dumbrepati et al. 2008; Djilali et al. 2012; Lee et al. 2013; Lino et al. 2018). Although, it has been shown that cane molasses have inhibitory effects in defined bioprocesses, especially citric acid fermentation (Chen et al. 2014), the bacteriostatic or bactericidal influences of cane molasses have been not reported. However, the inhibitory effects of cane molasses in such industrial bioprocesses is mainly attributed to the relatively high content of trace metals such as magnesium, zinc, manganese, calcium and iron, which interfere in metabolic pathways, and consequently, intervene the production of defined metabolites. Hence, cane molasses can be used as a promising substrate for production of the biomass of microbes, including LAB (Amorim et al. 2003; Barbosa et al. 2016). Considering the expenses of the nitrogen sources (beef extract, casein hydrolysate, and yeast extract) for efficient overproduction of the biomass of these fastidious microbes, a critical point for designing an effective medium seems to be the replacement of these expensive components with economic and efficient nitrogenous substrates (Horn et al. 2005; Manzoor et al. 2017). Furthermore, the comparative estimation of the expenses for the medium preparation have shed light on the possibility of further medium optimization (Djilali et al. 2012; Yoo et al. 2018).

The preliminary screening studies conducted to replace the costly beef extract and yeast extract with economic nitrogen sources. Strain RPR42 had significant growth efficiencies in CSL and WGE although the produced biomass was significantly less than those obtained in cane molasses and cheese whey (Additional file 1: Figures S1–S3). In comparison, the detectable growth of strain RPR42 in cheese whey (Additional file 1: Figure S3) and cane molasses (Figs. 1, 2, 3) showed the potential of these substrates to be used as the principal components of the medium. Such differences can be explained by the carbohydrate and organic nitrogen contents of these substrates (Dumbrepati et al. 2008; Djilali et al. 2012; Lee et al. 2013, Manzoor et al. 2017). Cane molasses normally has > 50% (v/v) carbohydrate content. Also, cane molasses contains significant growth factors like B-group vitamins (Dumbrepati et al. 2008; Teclu et al. 2009; Lee et al. 2013; Barbosa et al. 2016). However, it lacks a considerable protein, peptide and amino acid content to supply the needed nitrogen and growth factors for biomass overproduction of LAB strains (Amorim et al. 2003; Okafor and Okeke 2017). In place, the used cheese whey powder contained 76% (w/v) lactose content which is appropriate to supply the carbon and either energy for the growth of lactose-utilizing LAB strains (Manzoor et al. 2017). Additionally, cheese whey has reportedly significant concentrations of fats, protein and B-group vitamins, and so it can supply the growth factors which lactobacilli need for efficient growth (Siso 1996; Manzoor et al. 2017). Thus, cane molasses and cheese whey can supply the principal components (carbon and nitrogen sources) for overproduction of the biomass of LAB strains (Amorim et al. 2003; Lee et al. 2013; Manzoor et al. 2017). In comparison, CSL tend to be a suitable nitrogen source due to the considerable protein content. Also, it has a significant carbohydrate concentration (mainly as dextrin) (Cardinal and Hedrick 1948). However, our experiments showed that CSL has a lower efficiency than cheese whey to support the growth of strain RPR42 in the cane molasses-based media (Figs. 1, 2, 3, Tables 1 and 2). In addition to the chemical differences of CSL and cheese whey which affect their role in supporting the growth of strain RPR42, the acidic nature of CSL, which is a result of lactic acid content of this substrate, can be inhibitory for especial groups of microbes (including LAB) (Amorim et al. 2003). The next studied nitrogenous substrate was WGE, which had the minor effect on the growth of strain RPR42 in above media (Figs. 1, 2, 3, Tables 1 and 2). This substrate contains considerable quantities of carbohydrates, proteins and fat, which together with vitamins and minerals tend to be a suitable substrate which can support the growth of LAB strains (Brandolini and Hidalgo 2012), but our studies showed that this substrate has no detectable effect when used in the cane molasses-based media (Figs. 1, 2, 3, Tables 1 and 2).

A finding from the concomitant usage of multiple substrates was the fact that the addition of CSL, WGE and cheese whey with the cane molasses decreased the needed concentrations of all these components for biomass production of strain RPR42. Lee et al. (2013), have reported a similarly phenomenon in a medium optimization study aiming enhanced lactic acid production with combined cane molasses and CSL solutions. Lee et al. (2013) has explained this phenomenon as a reaction to higher osmolarities of the medium. The significant osmolant contents in CSL, cane molasses, WGE, and cheese whey can impact the growth of the microbial strains. Thus, the total sugar content of the assessed cane molasses-based media can affect the growth of strain RPR42.

Polysorbates increased the biomass production of strain RPR42 in various formula of cane molasses-based media. Such positive effects of polysorbates on the growth of lactobacilli seems to be associated to the fact that these fastidious firmicutes can utilize the fatty acid moieties of polysorbates in the biosynthetic pathways (Reitermayer et al. 2018). Furthermore, the effects of polysorbates on the growth of strain RPR42 were more detectable in cane molasses-based media containing CSL and WGE than cheese whey (Additional file 1: Figures S5, S7, and S9). Thus, the effectiveness of the polysorbates on the biomass production should depend on the medium formulation since the presence of CSL, WGE, and cheese whey influenced the positive effect and either the optimal concentration of polysorbates (Additional file 1: Figures S5, S7, and S9). Such an effect may be related to the different lipid composition of the above substrates (Brandolini and Hidalgo 2012).

As factorial design and either Box–Behnken design experiments highlighted, cheese whey significantly enhanced the growth of strain RPR42 in cane molasses-based media (Figs. 3 and 4). Hence, comparing the variables defined for the Box–Behnken design experiments, cheese whey with an F value of 708.34%, was found as the most affecting nitrogenous compound (Table 2, Fig. 4). Also, using combined formula of cheese whey and cane molasses resulted in a detectable enhancement of the produced biomass of strain RPR42 (Fig. 3). The detectable effect of cheese whey on the biomass production of strain RPR42 which was revealed in the Box–Behnken design experiments (Fig. 4), was in line with the results of the preliminary screenings (Fig. 3 and Additional file 1: Figure S3) and those of factorial design experiments (Figs. 1 and 4). Similarly, CSL increased the cell density of strain RPR42 in the cane molasses-based media (Table 1, Figs. 1 and 4). The experiments indicated that CSL can be regarded as a growth promoting nitrogenous substrate which can enhance the biomass production of strain RPR42 in cane molasses-based media (F value = 320.33%) (Table 1, Fig. 4). An important point for using CSL in the medium formulations was the acidic nature (pH ~ 4.0) of this complex syrup which is a result of its considerable lactic acid content (Cardinal and Hedrick 1948). The reaction of strain RPR42 to various CSL concentrations was firstly studied without pH adjustments and the growth yield of strain RPR42 in such solutions of CSL was not detectable (data not shown). Then, pH adjustments (pH = 6.8, NH_4_OH 25%) resulted in detectable growth of strain RPR42 in CSL solutions although the growth was not yet comparable to those observed in cane molasses and cheese whey solutions (Figs. 1 and 3). Such an acidity of CSL originates from the process through which CSL is manufactured. The thermo-tolerant LAB can apparently be enriched through this process which results in accumulation of a considerable amount of lactic acid (~ 26%) (Cardinal and Hedrick 1948). Similarly, strain RPR42 produced significant cell densities when grown in WGE as a whole medium and its growth was further enhanced in combined cane molasses + WGE media (Additional file 1: Figure S1). But, the influence of WGE on the growth of strain RPR42 in cane molasses + WGE media was not comparable to those observed in such media which contained cheese whey or CSL (Additional file 1: Figures S2 and S3). As Box–Behnken design experiments showed (Table 1, Fig. 4), the F value of WGE variable (59.17%) was very lower than those of cheese whey (708.34%) and CSL (320.33%) although its effect on the biomass production of strain RPR42 was still significant. Such an inferior effect of WGE was also observed in the preliminary screenings (Additional file 1: Figures S1–S3) and either factorial design experiments (Figs. 1, 2, 3).

It was also shown that the addition of the combined minerals to the cane molasses-based media containing CSL, WGE, and cheese whey had minor effects on the growth of strain RPR42 (Additional file 1: Tables S3–S5). Such a minor effect of these minerals have been also observed when the growth of strain RPR42 in cane molasses as a whole medium was assessed (data not shown). However, the inferior effect of the minerals can be associated to the mineral contents of the studied substrates.

The final optimization experiments performed in factorial (Additional file 1: Table S2) and either central composite designs (Tables 3 and 4) showed that a combined formula of nitrogenous substrates including CSL, WGE, cheese whey, and casein hydrolysate can promisingly increase the biomass production of strain RPR42 in a glucose-enriched cane molasses solution. Among these medium components, casein hydrolysate had the major effect and the minor influence was reported for glucose (Table 4). The suggested medium outcompeted the standard MRS medium when biomass production of strain RPR42 concerned and the efficiency of the medium was comparable to those reported in similar researches. For example, Horn et al. have reported a detectable cell density production of *L. plantarum* strain NC8 in a medium in which 5 g/L of peptones (prepared from hydrolysates of fish viscera) has been used in place of 22 g/L of beef extract, casein hydrolysate and yeast extract which are defined in the standard MRS medium. Similarly, Dumbrepati et al. have shown that the biomass production of a mutant strain of *L. delbrueckii* subsp. *delbrueckii* required insignificant yeast extract. It has been also indicated that the nitrogen content of the cane molasses is sufficient to support the growth of that LAB strain. Additionally, Krzywonos and Eberhard have reported a high cell density, 1.6 × 10^10^ CFU/mL), production of *L. plantarum* MiLAB 393 in an economic medium composed of 1.77 g/L of yeast extract, 10% molasses, sieved stillage (pH-adjusted, NH_4_OH). In comparison, our findings, on the growth requirements of strain RPR42 tend to be not in line with the observations reported in the above studies and such differences may be linked to intra-species varieties which have been observed in *Lactobacillus* species (Papizadeh et al. 2017).

Concerning the environmental issues regarding agricultural by-products, cane molasses and cheese whey together with CSL and WGE were used in this study to design an economic and efficient medium which can be used for industrial biomass production of LAB strains. Hence, environmentally hazardous wastes can be used for commercial manufacture of high value biological products.

## Supplementary information

**Additional file 1: Figure S1.** The biomass production of strain RPR42 in a gradient of CSL concentrations. The averages for the triplicate dry biomass weights are presented and finally values rounded to the nearest 0.00-0.09. Error bars represent the standard error. **Figure S2.** The biomass production of strain RPR42 in a gradient of WGE concentrations. The averages for the triplicate dry biomass weights are presented and finally values rounded to the nearest 0.00-0.09. Error bars represent the standard error. **Figure S3.** The biomass production of strain RPR42 in a gradient of Cheese whey concentrations. The averages for the triplicate dry biomass weights are presented and finally values rounded to the nearest 0.00-0.09. Error bars represent the standard error. **Figure S4.** Optimal concentration of polysorbates, Tween 20, 40, 60, and 80, in the presence of 10% and 7.5% (v/v) of cane molasses and CSL, respectively. The averages for the triplicate dry biomass weights are presented and finally values rounded to the nearest 0.00-0.09. Error bars represent the standard error. **Figure S5.** A factorial design for studying the influence of Tween 20, 40, 60, and 80 (each in a single level) on the biomass production of strain RPR42 in a medium in which the concentration of cane molasses and CSL were 10% and 7.5% (v/v), respectively. The averages for the triplicate dry biomass weights are presented and finally values rounded to the nearest 0.00-0.09. **Figure S6.** The influence of polysorbates (Tween 20, 40, 60, and 80) on the biomass production of strain RPR42 in the presence of cane molasses (15%) and WGE (70%). Error bars represent the standard error. **Figure S7.** The effects of the polysorbates, Tween 20, 40, 60, and 80 (each in a single level), on the biomass production of strain RPR42 in a factorial experiment. The concentration of cane molasses and WGE were 15% (v/v) and 70% (v/v), respectively. The averages for the triplicate dry biomass weights are presented and finally values rounded to the nearest 0.00-0.09. **Figure S8.** Optimal concentration of polysorbates; Tween 20, 40, 60, and 80 for the growth of strain RPR42 in the presence of cane molasses (10% (v/v)) and cheese whey (5% (w/v)). Error bars represent the standard error. **Figure S9.** The influence of the polysorbates; Tween 20, 40, 60, and 80 (each in a single level), on the biomass production of strain RPR42 in a factorial design. The concentration of cane molasses and cheese whey were 10% (v/v) and 5% (w/v), respectively. The averages for the triplicate dry biomass weights are presented and finally values rounded to the nearest 0.00-0.09. **Figure S10.** The influence of casein hydrolyzate and glucose on the growth of strain RPR42. The concentration of cane molasses was: 10% (v/v) in CSL-C, CSL-G, Whey-C and Whey-G; and it was 15% in WGE-C and WGE-G. CSL concentration was 7.5% (v/v) in CSL-C and CSL-G media. WGE concentration was 70% (v/v) in WGE-C and WGE-G media. Cheese whey concentration in Whey-C and Whey-G media was 5% (w/v). CSL-C, WGE-C, Whey-C contained casein hydrolysate and CSL-G, WGE-G and Whey-G contained glucose. Error bars represent the standard error. **Table S1.** The full factorial designs (4^2^) used for optimization of the nitrogenous sources and cane molasses solutions for biomass production of strain RPR42. The concentrations of all ingredients were in percent (cane molasses and CSL; (v/v), Cheese whey; (w/v)). **Table S2.** Partial factorial design for screening the effects of glucose, casein hydrolysate, cane molasses, CSL, WGE, cheese whey, and polysorbates (A; Casein hydrolysate, B; Cane molasses; C; CSL, D; WGE, E; Cheese whey, F; Glucose, G; 1:1:1 mixture of Tween 20, 60, and 80 polysorbates). **Table S3.** The influence of the five mineral components (each in a single level) on the biomass production of strain RPR42 in a factorial experiment design. The cane molasses and CSL concentrations were 15% and 7.5% (v/v), respectively. Concentration (g/L) of the minerals were: 4; di-ammonium citrate, 7; sodium acetate, 5; K_2_HPO_4_, 0.3; MgSO_4_, and 0.05; MnSO_4_. The averages for the triplicate dry biomass weights are presented and finally values rounded to the nearest 0.00-0.09. **Table S4.** Factorial design experiment screening the influence of the five mineral components (each in a single level) on the biomass production of strain RPR42. The concentration of cane molasses and WGE were 15% and 70% (v/v). The concentration of cane molasses and cheese whey were 10% and 5% (v/v), respectively. Concentration (g/L) of the minerals were: 4; di-ammonium citrate, 7; sodium acetate, 5; K_2_HPO_4_, 0.3; MgSO_4_, and 0.05; MnSO_4_. The averages for the triplicate dry biomass weights are presented and finally values rounded to the nearest 0.00-0.09. **Table S5.** Factorial design for assessment of the effects of mineral components (each in a single level) on the biomass production of strain RPR42 in a medium solution in which the concentration of cane molasses and cheese whey were 10% and 5% (w/v), respectively. Concentration (g/L) of the minerals were: 4; di-ammonium citrate, 7; sodium acetate, 5; K_2_HPO_4_, 0.3; MgSO_4_, and 0.05; MnSO_4_. The averages for the triplicate dry biomass weights are presented and finally values rounded to the nearest 0.00-0.09.

## Data Availability

Not applicable.
