# Supplementary material for: Screening for efficient nitrogen sources for overproduction of the biomass of the functionally probiotic L. plantarum strain RPR42 in a cane molasses-based medium
Source: AMB Express. 2020 Mar 17;10:53. doi: 10.1186/s13568-020-00976-x (PMC7078425; doi:10.1186/s13568-020-00976-x)
Supplement: Supplementary file 1 — Additional file 1: Figure S1. The biomass production of strain RPR42 in a gradient of CSL concentrations. The averages for the triplicate dry biomass weights are presented and finally values rounded to the nearest 0.00-0.09. Error bars represent the standard error. Figure S2. The biomass production of strain RPR42 in a gradient of WGE concentrations. The averages for the triplicate dry biomass weights are presented and finally values rounded to the nearest 0.00-0.09. Error bars represent the standard error. Figure S3. The biomass production of strain RPR42 in a gradient of Cheese whey concentrations. The averages for the triplicate dry biomass weights are presented and finally values rounded to the nearest 0.00-0.09. Error bars represent the standard error. Figure S4. Optimal concentration of polysorbates, Tween 20, 40, 60, and 80, in the presence of 10% and 7.5% (v/v) of cane molasses and CSL, respectively. The averages for the triplicate dry biomass weights are presented and finally values rounded to the nearest 0.00-0.09. Error bars represent the standard error. Figure S5. A factorial design for studying the influence of Tween 20, 40, 60, and 80 (each in a single level) on the biomass production of strain RPR42 in a medium in which the concentration of cane molasses and CSL were 10% and 7.5% (v/v), respectively. The averages for the triplicate dry biomass weights are presented and finally values rounded to the nearest 0.00-0.09. Figure S6. The influence of polysorbates (Tween 20, 40, 60, and 80) on the biomass production of strain RPR42 in the presence of cane molasses (15%) and WGE (70%). Error bars represent the standard error. Figure S7. The effects of the polysorbates, Tween 20, 40, 60, and 80 (each in a single level), on the biomass production of strain RPR42 in a factorial experiment. The concentration of cane molasses and WGE were 15% (v/v) and 70% (v/v), respectively. The averages for the triplicate dry biomass weights are presented and finally values rou [file 13568_2020_976_MOESM1_ESM.docx]

Additional data

**Figure S1** The biomass production of strain RPR42 in a gradient of CSL concentrations. The averages for the triplicate dry biomass weights are presented and finally values rounded to the nearest 0.00-0.09. Error bars represent the standard error.

**Figure S2** The biomass production of strain RPR42 in a gradient of WGE concentrations. The averages for the triplicate dry biomass weights are presented and finally values rounded to the nearest 0.00-0.09. Error bars represent the standard error.

**Figure S3** The biomass production of strain RPR42 in a gradient of Cheese whey concentrations. The averages for the triplicate dry biomass weights are presented and finally values rounded to the nearest 0.00-0.09. Error bars represent the standard error.

**Figure S4** Optimal concentration of polysorbates, Tween 20, 40, 60, and 80, in the presence of 10% and 7.5% (v/v) of cane molasses and CSL, respectively. The averages for the triplicate dry biomass weights are presented and finally values rounded to the nearest 0.00-0.09. Error bars represent the standard error.

**Figure S5** A factorial design for studying the influence of Tween 20, 40, 60, and 80 (each in a single level) on the biomass production of strain RPR42 in a medium in which the concentration of cane molasses and CSL were 10% and 7.5% (v/v), respectively. The averages for the triplicate dry biomass weights are presented and finally values rounded to the nearest 0.00-0.09.

**Figure S6** The influence of polysorbates (Tween 20, 40, 60, and 80) on the biomass production of strain RPR42 in the presence of cane molasses (15%) and WGE (70%). Error bars represent the standard error.

**Figure S7** The effects of the polysorbates, Tween 20, 40, 60, and 80 (each in a single level), on the biomass production of strain RPR42 in a factorial experiment. The concentration of cane molasses and WGE were 15% (v/v) and 70% (v/v), respectively. The averages for the triplicate dry biomass weights are presented and finally values rounded to the nearest 0.00-0.09.

**Figure S8** Optimal concentration of polysorbates; Tween 20, 40, 60, and 80 for the growth of strain RPR42 in the presence of cane molasses (10% (v/v)) and cheese whey (5% (w/v)). Error bars represent the standard error.

**Figure S9** The influence of the polysorbates; Tween 20, 40, 60, and 80 (each in a single level), on the biomass production of strain RPR42 in a factorial design. The concentration of cane molasses and cheese whey were 10% (v/v) and 5% (w/v), respectively. The averages for the triplicate dry biomass weights are presented and finally values rounded to the nearest 0.00-0.09.

**Figure S10** The influence of casein hydrolyzate and glucose on the growth of strain RPR42. The concentration of cane molasses was: 10% (v/v) in CSL-C, CSL-G, Whey-C and Whey-G; and it was 15% in WGE-C and WGE-G. CSL concentration was 7.5% (v/v) in CSL-C and CSL-G media. WGE concentration was 70% (v/v) in WGE-C and WGE-G media. Cheese whey concentration in Whey-C and Whey-G media was 5% (w/v). CSL-C, WGE-C, Whey-C contained casein hydrolysate and CSL-G, WGE-G and Whey-G contained glucose. Error bars represent the standard error.

**Table S1** The full factorial designs (4^2^) used for optimization of the nitrogenous sources and cane molasses solutions for biomass production of strain RPR42. The concentrations of all ingredients were in percent (cane molasses and CSL; (v/v), Cheese whey; (w/v)).

| **Factorial design 1** | | |
| --- | --- | --- |
| **Treatment** | **Cane molasses** | **CSL** |
| T1 | 25 | 7.5 |
| T2 | 25 | 10 |
| T3 | 25 | 12.5 |
| T4 | 25 | 15 |
| T5 | 20 | 7.5 |
| T6 | 20 | 10 |
| T7 | 20 | 12.5 |
| T8 | 20 | 15 |
| T9 | 15 | 7.5 |
| T10 | 15 | 10 |
| T11 | 15 | 12.5 |
| T12 | 15 | 15 |
| T13 | 12.5 | 7.5 |
| T14 | 12.5 | 10 |
| T15 | 12.5 | 12.5 |
| T16 | 12.5 | 15 |
| **Factorial design 2** | | |
| **Treatment** | **Cane molasses** | **WGE** |
| T1 | 25 | 40 |
| T2 | 25 | 50 |
| T3 | 25 | 60 |
| T4 | 25 | 70 |
| T5 | 20 | 40 |
| T6 | 20 | 50 |
| T7 | 20 | 60 |
| T8 | 20 | 70 |
| T9 | 15 | 40 |
| T10 | 15 | 50 |
| T11 | 15 | 60 |
| T12 | 15 | 70 |
| T13 | 12.5 | 40 |
| T14 | 12.5 | 50 |
| T15 | 12.5 | 60 |
| T16 | 12.5 | 70 |
| **Factorial design 3** | | |
| **Treatment** | **Cane molasses** | **Cheese whey** |
| T1 | 25 | 0.5 |
| T2 | 25 | 2.5 |
| T3 | 25 | 5 |
| T4 | 25 | 10 |
| T5 | 20 | 0.5 |
| T6 | 20 | 2.5 |
| T7 | 20 | 5 |
| T8 | 20 | 10 |
| T9 | 15 | 0.5 |
| T10 | 15 | 2.5 |
| T11 | 15 | 5 |
| T12 | 15 | 10 |
| T13 | 12.5 | 0.5 |
| T14 | 12.5 | 2.5 |
| T15 | 12.5 | 5 |
| T16 | 12.5 | 10 |
| T17 | 10 | 0.5 |
| T18 | 10 | 2.5 |
| T19 | 10 | 5 |
| T20 | 10 | 10 |
| T21 | 7.5 | 0.5 |
| T22 | 7.5 | 2.5 |
| T23 | 7.5 | 5 |
| T24 | 7.5 | 10 |
| T25 | 5 | 0.5 |
| T26 | 5 | 2.5 |
| T27 | 5 | 5 |
| T28 | 5 | 10 |

**Table S2** Partial factorial design for screening the effects of glucose, casein hydrolysate, cane molasses, CSL, WGE, cheese whey, and polysorbates (A; Casein hydrolysate, B; Cane molasses; C; CSL, D; WGE, E; Cheese whey, F; Glucose, G; 1:1:1 mixture of Tween 20, 60, and 80 polysorbates).

| **A: Casein hydrolysate (g/L)** | **B: Cane Molasses (%)** | **C: CSL (%)** | **D: WGE (%)** | **E: Cheese Whey (%)** | **F: Glucose (g/L)** | **G: Polysorbate (%)** | **Dry Biomass (g/L)** |
| --- | --- | --- | --- | --- | --- | --- | --- |
| 4.5 | 10 | 3.5 | 38.1 | 2.6 | 0 | 0 | 11.39 |
| 0 | 10 | 3.5 | 38.1 | 2.6 | 0 | 0.07 | 9.81 |
| 0 | 10 | 3.5 | 38.1 | 2.6 | 0 | 0 | 9.68 |
| 4.5 | 10 | 3.5 | 38.1 | 2.6 | 2.5 | 0 | 11.65 |
| 4.5 | 10 | 3.5 | 38.1 | 2.6 | 2.5 | 0.05 | 11.83 |
| 4.5 | 10 | 3.5 | 38.1 | 2.6 | 2.5 | 0.07 | 11.84 |
| 0 | 10 | 3.5 | 38.1 | 2.6 | 2.5 | 0.07 | 10.66 |
| 4.5 | 10 | 3.5 | 38.1 | 2.6 | 0 | 0.07 | 11.65 |
| 4.5 | 10 | 3.5 | 0 | 2.6 | 2.5 | 0.07 | 11.77 |
| 4.5 | 10 | 0 | 38.1 | 2.6 | 2.5 | 0.07 | 11.14 |
| 4.5 | 10 | 3.5 | 38.1 | 0 | 2.5 | 0.07 | 10.93 |

**Table S3** The influence of the five mineral components (each in a single level) on the biomass production of strain RPR42 in a factorial experiment design. The cane molasses and CSL concentrations were 15% and 7.5% (v/v), respectively. Concentration (g/L) of the minerals were: 4; di-ammonium citrate, 7; sodium acetate, 5; K_2_HPO_4_, 0.3; MgSO_4_, and 0.05; MnSO_4_. The averages for the triplicate dry biomass weights are presented and finally values rounded to the nearest 0.00-0.09.

| Run number | K_2_HPO_4_ | MgSO_4_ | MnSO_4_ | Sodium acetate | Di ammonium citrate | Dry Biomass (g/L) |
| --- | --- | --- | --- | --- | --- | --- |
| 1 | ■ | ■ |  |  |  | 8.65 |
| 2 | ■ |  | ■ |  |  | 8.63 |
| 3 | ■ |  |  | ■ |  | 8.65 |
| 4 | ■ |  |  |  | ■ | 8.64 |
| 5 | ■ | ■ | ■ |  |  | 8.62 |
| 6 | ■ | ■ |  | ■ |  | 8.66 |
| 7 | ■ | ■ |  |  | ■ | 8.68 |
| 8 | ■ |  | ■ | ■ |  | 8.66 |
| 9 | ■ |  | ■ |  | ■ | 8.67 |
| 10 | ■ |  |  | ■ | ■ | 8.7 |
| 11 | ■ | ■ |  | ■ | ■ | 8.7 |
| 12 | ■ |  | ■ | ■ | ■ | 8.7 |
| 13 | ■ | ■ | ■ | ■ |  | 8.66 |
| 14 | ■ | ■ | ■ |  | ■ | 8.67 |
| 15 | ■ | ■ | ■ | ■ | ■ | 8.7 |
| 16 |  | ■ | ■ |  |  | 8.6 |
| 17 |  | ■ |  | ■ |  | 8.61 |
| 18 |  | ■ |  |  | ■ | 8.61 |
| 19 |  | ■ | ■ | ■ |  | 8.61 |
| 20 |  | ■ | ■ |  | ■ | 8.61 |
| 21 |  | ■ | ■ | ■ | ■ | 8.66 |
| 22 |  | ■ |  | ■ | ■ | 8.65 |
| 23 |  |  | ■ | ■ |  | 8.63 |
| 24 |  |  | ■ |  | ■ | 8.64 |
| 25 |  |  |  | ■ | ■ | 8.64 |

**Table S4** Factorial design experiment screening the influence of the five mineral components (each in a single level) on the biomass production of strain RPR42. The concentration of cane molasses and WGE were 15% and 70% (v/v). The concentration of cane molasses and cheese whey were 10% and 5% (v/v), respectively. Concentration (g/L) of the minerals were: 4; di-ammonium citrate, 7; sodium acetate, 5; K_2_HPO_4_, 0.3; MgSO_4_, and 0.05; MnSO_4_. The averages for the triplicate dry biomass weights are presented and finally values rounded to the nearest 0.00-0.09.

| Run number | K_2_HPO_4_ | MgSO_4_ | MnSO_4_ | Sodium Acetate | Di ammonium citrate | Dry Biomass (g/L) |
| --- | --- | --- | --- | --- | --- | --- |
| 1 | ■ | ■ |  |  |  | 7.64 |
| 2 | ■ |  | ■ |  |  | 7.64 |
| 3 | ■ |  |  | ■ |  | 7.64 |
| 4 | ■ |  |  |  | ■ | 7.65 |
| 5 | ■ | ■ | ■ |  |  | 7.66 |
| 6 | ■ | ■ |  | ■ |  | 7.68 |
| 7 | ■ | ■ |  |  | ■ | 7.67 |
| 8 | ■ |  | ■ | ■ |  | 7.65 |
| 9 | ■ |  | ■ |  | ■ | 7.68 |
| 10 | ■ |  |  | ■ | ■ | 7.7 |
| 11 | ■ | ■ |  | ■ | ■ | 7.73 |
| 12 | ■ |  | ■ | ■ | ■ | 7.74 |
| 13 | ■ | ■ | ■ | ■ |  | 7.73 |
| 14 | ■ | ■ | ■ |  | ■ | 7.74 |
| 15 | ■ | ■ | ■ | ■ | ■ | 7.76 |
| 16 |  | ■ | ■ |  |  | 7.64 |
| 17 |  | ■ |  | ■ |  | 7.64 |
| 18 |  | ■ |  |  | ■ | 7.64 |
| 19 |  | ■ | ■ | ■ |  | 7.68 |
| 20 |  | ■ | ■ |  | ■ | 7.69 |
| 21 |  | ■ | ■ | ■ | ■ | 7.72 |
| 22 |  | ■ |  | ■ | ■ | 7.67 |
| 23 |  |  | ■ | ■ |  | 7.65 |
| 24 |  |  | ■ |  | ■ | 7.66 |
| 25 |  |  |  | ■ | ■ | 7.68 |

**Table S5** Factorial design for assessment of the effects of mineral components (each in a single level) on the biomass production of strain RPR42 in a medium solution in which the concentration of cane molasses and cheese whey were 10% and 5% (w/v), respectively. Concentration (g/L) of the minerals were: 4; di-ammonium citrate, 7; sodium acetate, 5; K_2_HPO_4_, 0.3; MgSO_4_, and 0.05; MnSO_4_. The averages for the triplicate dry biomass weights are presented and finally values rounded to the nearest 0.00-0.09.

| Run number | K_2_HPO_4_ | MgSO_4_ | MnSO_4_ | Sodium Acetate | Di ammonium citrate | Dry Biomass (g/L) |
| --- | --- | --- | --- | --- | --- | --- |
| 1 | ■ | ■ |  |  |  | 9.33 |
| 2 | ■ |  | ■ |  |  | 9.33 |
| 3 | ■ |  |  | ■ |  | 9.33 |
| 4 | ■ |  |  |  | ■ | 9.33 |
| 5 | ■ | ■ | ■ |  |  | 9.33 |
| 6 | ■ | ■ |  | ■ |  | 9.36 |
| 7 | ■ | ■ |  |  | ■ | 9.36 |
| 8 | ■ |  | ■ | ■ |  | 9.36 |
| 9 | ■ |  | ■ |  | ■ | 9.36 |
| 10 | ■ |  |  | ■ | ■ | 9.36 |
| 11 | ■ | ■ |  | ■ | ■ | 9.37 |
| 12 | ■ |  | ■ | ■ | ■ | 9.37 |
| 13 | ■ | ■ | ■ | ■ |  | 9.37 |
| 14 | ■ | ■ | ■ |  | ■ | 9.37 |
| 15 | ■ | ■ | ■ | ■ | ■ | 9.37 |
| 16 |  | ■ | ■ |  |  | 9.29 |
| 17 |  | ■ |  | ■ |  | 9.3 |
| 18 |  | ■ |  |  | ■ | 9.28 |
| 19 |  | ■ | ■ | ■ |  | 9.32 |
| 20 |  | ■ | ■ |  | ■ | 9.32 |
| 21 |  | ■ | ■ | ■ | ■ | 9.32 |
| 22 |  | ■ |  | ■ | ■ | 9.32 |
| 23 |  |  | ■ | ■ |  | 9.32 |
| 24 |  |  | ■ |  | ■ | 9.31 |
| 25 |  |  |  | ■ | ■ | 9.31 |
